# The role of the complement system in kidney glomerular capillary thrombosis

**DOI:** 10.3389/fimmu.2022.981375

**Published:** 2022-09-14

**Authors:** Yoko Yoshida, Hiroshi Nishi

**Affiliations:** Division of Nephrology and Endocrinology, The University of Tokyo Graduate School of Medicine, Tokyo, Japan

**Keywords:** complement, kidney, platelet, neutrophil, atypical hemolytic uremic syndrome, thrombosis, lupus nephritis, coagulation

## Abstract

The complement system is part of the innate immune system. The crucial step in activating the complement system is the generation and regulation of C3 convertase complexes, which are needed to generate opsonins that promote phagocytosis, to generate C3a that regulates inflammation, and to initiate the lytic terminal pathway through the generation and activity of C5 convertases. A growing body of evidence has highlighted the interplay between the complement system, coagulation system, platelets, neutrophils, and endothelial cells. The kidneys are highly susceptible to complement-mediated injury in several genetic, infectious, and autoimmune diseases. Atypical hemolytic uremic syndrome (aHUS) and lupus nephritis (LN) are both characterized by thrombosis in the glomerular capillaries of the kidneys. In aHUS, congenital or acquired defects in complement regulators may trigger platelet aggregation and activation, resulting in the formation of platelet-rich thrombi in the kidneys. Because glomerular vasculopathy is usually noted with immunoglobulin and complement accumulation in LN, complement-mediated activation of tissue factors could partly explain the autoimmune mechanism of thrombosis. Thus, kidney glomerular capillary thrombosis is mediated by complement dysregulation and may also be associated with complement overactivation. Further investigation is required to clarify the interaction between these vascular components and develop specific therapeutic approaches.

## Introduction

### Complement system

**Figure 1:**
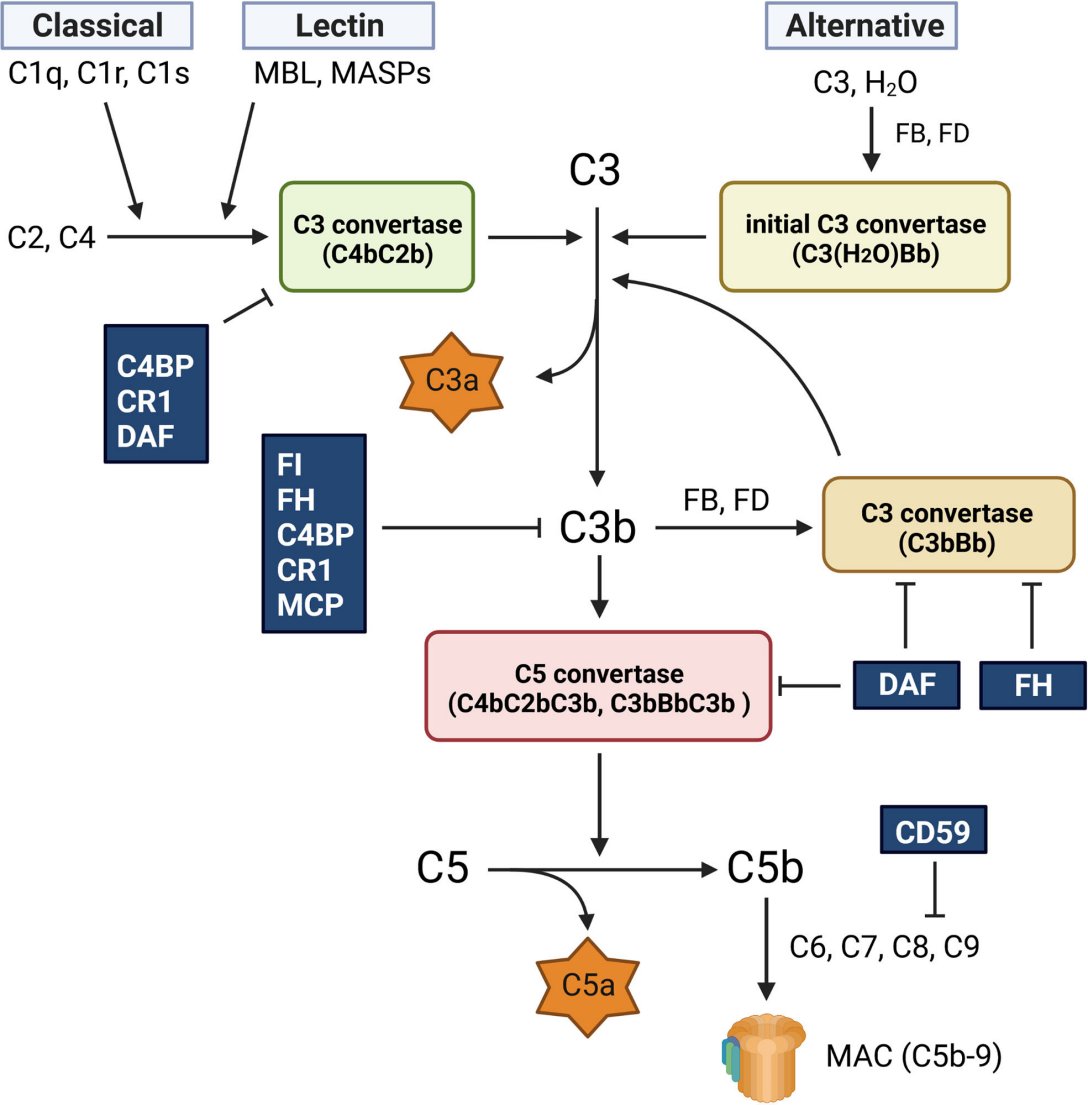
Complement activation pathway. The complement system is activated *via* three different pathways: classical (CP), lectin (LP), and alternative (AP). The CP is activated by the binding of C1q to antigen-bound antibodies. This reaction activates C1s and C1r, leading to the formation of the CP C3 convertase (C4bC2b). The LP generates the same C3 convertase as the CP, but its activation is caused by mannose-binding lectin (MBL) and MBL-associated serine proteases (MASPs). The AP is spontaneously activated *via* hydrolysis of C3 (C3 (H_2_O)), which generates the initial C3 convertase (C3(H_2_O)Bb). All three activation routes merge at the cleavage of C3 and lead to the formation of the C5 convertases (C4bC2bC3b and C3bBbC3b), which cleave C5 into C5a and C5b. The C5b fragment forms the membrane attack complex (C5b-9, MAC) by binding to C6, C7, C8, and C9. C9 polymerization is required for C5b-9 generation. C5b-9 creates pores in the membrane and lyses the target cells. The fragment of C3b is opsonin, and C3a and C5a are also known as anaphylatoxins and chemotactic factors, respectively.

The complement system is essential for the innate immune system to eliminate invading pathogens. The crucial step in activating the complement system is the generation and regulation of C3 convertase. All complement mechanisms in immune defense, namely opsonization, phagocytosis, inflammation, and target cell lysis, rely on the enzymatic step that generates C3 convertase. The complement system is activated by three different pathways: the classical (CP), lectin (LP), and alternative (AP) pathways (**Figure 1**). Although each of these routes has a different activation mechanism, they all produce C3 convertase, which cleaves C3 into C3a and C3b to activate the terminal complement pathway and generate C5b-9 (1).

The CP is activated by C1q recognition of antigen-bound antibodies, which eventually induces C1s to produce the CP C3 convertase (C4bC2b) through the cleavage of C2 and C4. In the LP, the binding of mannose-binding lectin (MBL) to microbial carbohydrates triggers the generation of C3 convertase (C4bC2b) *via* the MBL-associated serine proteases 1 and 2 (MASP-1 and MASP-2). In contrast, the AP is constitutively activated at low levels by the hydrolysis of C3 (C3(H_2_O)). C3(H_2_O) rapidly reacts with complement factors B (FB) and D, resulting in the formation of an initial fluid-phase C3 convertase (C3(H_2_O)Bb). This initial convertase can cleave C3 and generate C3b, which is generally inactivated by various complement regulatory proteins. However, in the presence of pathogens, C3b binds to the target surface and induces the formation of C3 convertase (C3bBb). All C3 convertases (CP/LP C4bC2b and AP C3bBb) can attach to the C3b fragment and form C5 convertases (CP/LP C4bC2bC3b and AP C3bBbC3b), which cleave C5 into C5a and C5b. The C5b molecule sequentially binds to C6, C7, and C8 to form C5b-8. Finally, C5b-8 binds to C9, which polymerizes and forms a transmembrane ring, leading to the formation of C5b-9, which is also known as the membrane attack complex (MAC).

Although cell lysis by C5b-9 is effective against gram-negative bacteria, the complement system also acts against gram-positive bacteria by promoting opsonization and neutrophil phagocytosis. The C3b and C4b fragments are opsonins, and opsonized pathogens are recognized by complement receptor type 1 (CR1) on neutrophils, which then phagocytose them. In addition to opsonization, complement C3b enhances antibody generation by B cells, and another important role of complement is the generation of two anaphylatoxins, C3a and C5a. These peptides support inflammation and activate cells expressing anaphylatoxin receptors (1).

### Negative regulators of the complement system and their expression in the kidneys

Various complement-regulatory proteins tightly control complement activation to protect autologous tissues from complement attack (2–4). The plasma protein C1 inhibitor (C1-INH) prevents the initiation of the CP and LP by binding to and inactivating C1r, C1s, and MASPs (5). During C3 convertase formation, various complement regulators function as cofactors for factor I (FI), a serine protease that inactivates C3b and C4b. Some regulators also exhibit decay acceleration activity, which decreases the stability of C3 convertases by accelerating the dissociation of Bb from C3bBb and/or C2b from C4bC2b (1, 2, 4).

Factor H (FH) and C4b-binding protein (C4BP) are fluid-phase proteins associated with FI-mediated C3b or C4b cleavage, as well as the decay acceleration activity of the AP or CP C3 convertase (1, 2, 4). Cell membrane inhibitors, such as CR1 and membrane cofactor protein (MCP), also function as cofactors for the inactivation of C3b and C4b *via* FI. CR1 also shows decay acceleration activity with respect to the CP C3 convertase, but MCP does not. Decay accelerating factor (DAF or CD55) and CD59 are glycosylphosphatidylinositol (GPI)-anchored membrane inhibitors of the complement system. As its name implies, DAF accelerates the decay of C3/C5 convertases, and CD59 inhibits C5b-9 formation by binding to C8 and C9. CR1-related gene/-protein y (Crry) is a rodent-specific membrane regulator with both cofactor and decay-accelerating activities, and is similar to human MCP and DAF (6). Animal experiments using Crry-neutralizing antibodies have revealed that Crry is a critical complement regulator in rodent kidneys (2).

In the context of renal physiology, MCP, CR1, DAF, and CD59 are expressed in the glomeruli and protect them from complement attacks. The expression levels of these regulatory proteins can be altered by complement attack and in various glomerular disorders (2). MCP, DAF, and CD59 are ubiquitously expressed in all resident glomerular cells, although DAF is barely detectable in the glomerular cells of normal kidneys (2). The expression of MCP and DAF, but not that of CD59, is concentrated in the juxtaglomerular apparatus (3). In contrast, the expression of CR1 is mainly restricted to podocytes (2).

Although FH is a fluid phase complement regulatory protein, it is also present on the cell surface. The C-terminal region of FH can bind to glycosaminoglycans and sialic acid on the cell surface. After binding to the cell surface, it can trap deposited C3b to induce FI-mediated C3b inactivation (4). Clinically, FH dysfunction is highly associated with renal impairment caused by atypical hemolytic uremic syndrome (aHUS) and C3 glomerulopathy, suggesting that FH-mediated complement regulation plays an essential role in kidney health.

## Roles of the complement system and inflammation in thrombosis

**Figure 2:**
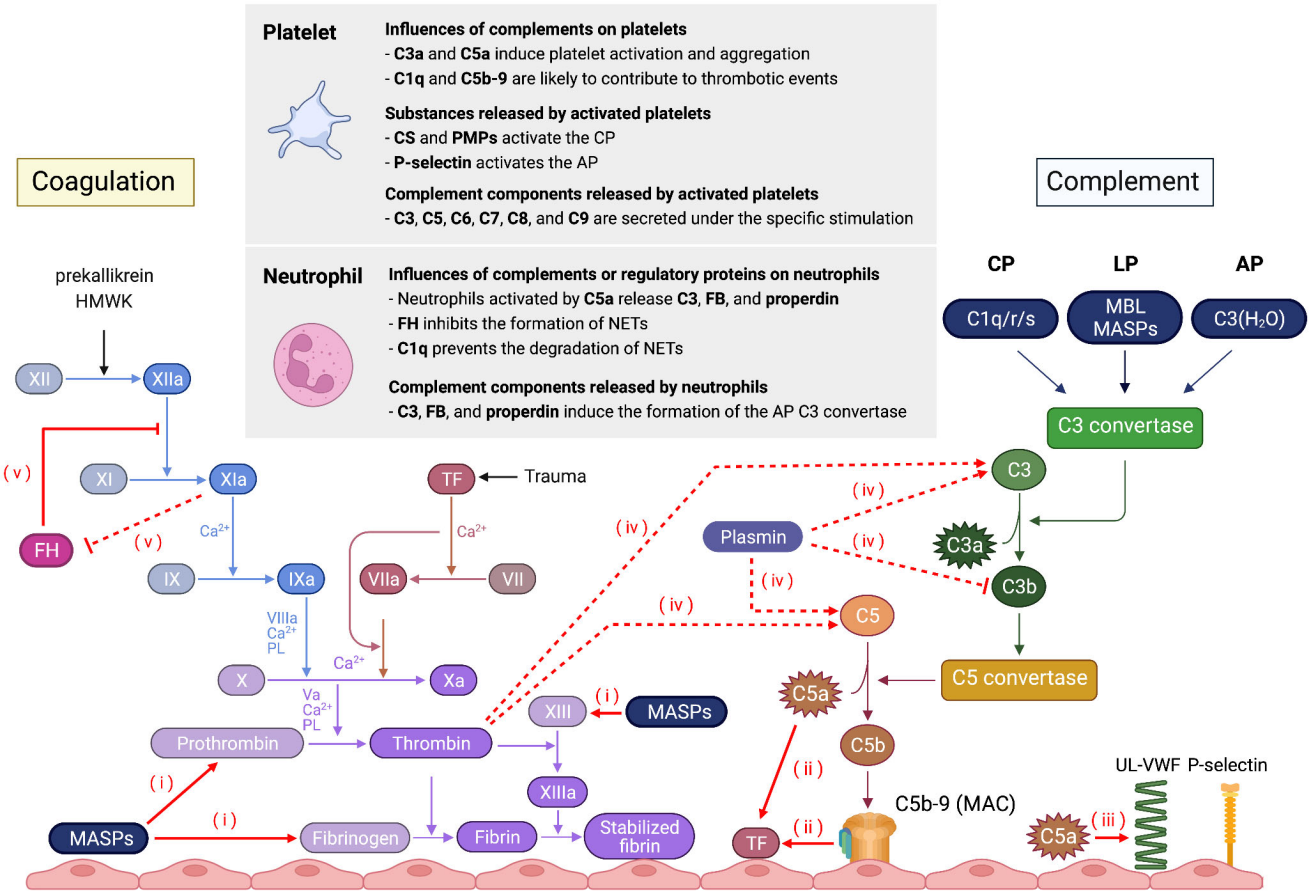
Schematic overview of the interaction between the complement system and the coagulation system, platelets, and neutrophils. (i) MASPs can cleave coagulation factors, such as prothrombin, fibrinogen, and factor XIII. (ii) C5a and C5b-9 enhance blood thrombogenicity through upregulation of TF in endothelial cells and neutrophils. (iii) C5a induces UL-VWF secretion and P-selectin expression. Conversely, coagulation factors activate the complement cascade. (iv) Thrombin and plasmin can generate C3a and C5a, whereas plasminogen enhances FI-mediated C3b cleavage in the presence of FH and plasmin degrades C3b. (v) Factor XIa decreases the cofactor and decay acceleration activity of FH and the ability binding of FH to bind to human endothelial cells. Conversely, FH inhibits FXIa activation.

The complement system, coagulation-fibrinolytic system, platelets, and leukocytes all form a close network and interact with each other (7). Therefore, dysregulation of any component can lead to multiple diseases with different pathological conditions and clinical manifestations (8) (**Figure 2**).

### Complement and coagulation systems

Both the complement and coagulation systems share common cascade pathways that involve proteolysis and enable an inflammatory response as a way of defending the host (9–11).

The complement system modulates the coagulation cascade in several ways. Both C5- and C3-deficient mice have longer tail bleeding time and reduced susceptibility to thrombosis, indicating that the complement system plays an essential role in the coagulation process (12). MASP-1 and MASP-2 cleave coagulation factors such as prothrombin, fibrinogen, factor XIII, and thrombin-activatable fibrinolysis inhibitors *in vitro* (13, 14). C5a and C5b-9 induce tissue factor (TF) expression, which initiates the extrinsic coagulation pathway in both endothelial cells and neutrophils (9, 15). C5a also induces the secretion of ultra large von Willebrand factor multimers and P-selectin and increases neutrophil adhesion to cultured endothelial cells (16). These suggest that C5a is an important inflammatory mediator between neutrophils and endothelial cells during the acute inflammatory response.

Coagulation factors can also activate the complement cascade at different levels. Thrombin cleaves C3 and C5 into C3a and C5a *in vitro*, which amplifies the activation of the complement system and the induction of chemotaxis and neutrophil activation (17, 18). Plasmin also cleaves C3 and C5 (17). Plasminogen, on the other hand, enhances FI-mediated cleavage of C3b in the presence of FH, and plasmin degrades C3b (19). Factor XIa (FXIa) and FH modulate each other. FXIa cleaves FH, which decreases the cofactor and decay acceleration activity of FH and the ability of FH to bind to human endothelial cells. Conversely, FH inhibits FXIa activation *via* either thrombin or factor XIIa (20).

### Complement system and platelets

Various complement components specifically activate platelets (10, 11). Thrombin-mediated platelet aggregation and release are enhanced by several complement components, such as C3, C5, C6, C7, C8, and C9 (21). Most of these proteins are stored in platelets and are secreted following activation (22–24). The binding of C1q to the C1q receptor (gC1qR/p33) expressed on platelets activates glycoprotein (GP) IIb-IIIa fibrinogen binding sites and P-selectin expression, which contributes to the thrombotic events associated with complement activation (25). The binding of C5b-9 induces a change in the membrane potential of platelets, thereby exposing the binding sites to factor Va and serving as a basis for the proteolytic generation of thrombin (26). In addition, anaphylatoxins C3a and C5a induce platelet activation and aggregation (27).

Platelets also release various molecules that activate or modulate the complement system (10, 11). Chondroitin sulfate released by thrombin-activated platelets induces fluid-phase activation of the CP in a C1q-dependent manner (28). Platelet microparticles also support the activation of the CP, whereas they induce the expression of C1-INH from the α-granules of platelets (29). Intriguingly, P-selectin expressed in activated platelets acts as a receptor for C3b, thereby initiating the activation of the AP (30). Experimental animal data have shown that C3, but not C5, is not redundant in platelet activation (12).

Furthermore, platelets have a complement regulatory system that limits complement activation on their surfaces. Platelets express MCP, CD55, and CD59 and carry C1-INH and FH (31, 32) in their α granules. In addition, activated platelets and neutrophils can remove C5b-9 in the form of microparticles (33). The absence or impairment of such regulatory proteins is associated with platelet dysfunction, alterations in platelet activation, or thrombocytopenia (8).

### Complement system, leukocytes, and the endothelium

Neutrophils accumulate at inflamed sites with the help of the chemoattractant C5a and phagocytose bacteria with surface deposits of C3b or iC3b *via* CR1 and CR3 (34, 35). Neutrophil extracellular traps (NETs) are also considered part of the human innate immune system. These form when neutrophils respond to bacteria or immune complexes by ejecting nuclear chromatin and digestive enzymes to kill pathogens (36) and can sometimes evoke autoimmune tissue injury (37, 38).

Properdin is a positive complement regulator that stabilizes C3 convertase in the AP (39). Intriguingly, activated neutrophils (including those activated by C5a) release C3, FB, and properdin (40), and induce the formation of AP C3 convertase, leading to C5a generation. C5a activates additional neutrophils, which secrete key components of the AP. C3- and C3a receptor (C3aR)-deficient knockout mice fail to form NETs (41, 42), suggesting that the complement system also affects the formation of NETs. Furthermore, C3b opsonization promoted the release of NETs (43). In addition, neutrophils stimulated with phorbol myristate acetate (PMA) secrete properdin and deposit it on NETs and certain bacteria to induce the formation of C5b–9 (40).

FH, a negative regulator of the AP, binds to neutrophils, inhibits the formation of PMA-stimulated NETs (44), and reduces the inflammatory response (45). Conversely, binding of C1q prevents the degradation of NETs by directly inhibiting DNAse-I by C1q (46). NETs also exert thrombogenic activity through their expression of functionally active TF (47), which is disrupted by complement C3 inhibition (48).

## Role of the complement system in glomerular capillary thrombosis in kidney disorders

### Thrombotic microangiopathy

Thrombotic microangiopathy (TMA) is a pathological condition caused by the formation of microvascular thrombi that leads to thrombocytopenia, microangiopathic hemolytic anemia, and end-organ damage (49). TMA is caused by various hereditary or acquired factors, and is classified into four main categories: thrombotic thrombocytopenic purpura (TTP), hemolytic uremic syndrome (HUS) caused by Shiga toxin-producing *Escherichia coli* (STEC), atypical HUS (aHUS), and secondary TMA.

TTP is caused by a severe deficiency of ADAMTS13 (a disintegrin-like metalloprotease with thrombospondin type 1 motif, member 13) resulting from genetic or acquired defects (50). STEC-induced HUS is predominantly found in children and is diagnosed based on the direct detection of Shiga toxins in feces and the presence of anti-lipopolysaccharide immunoglobulin M antibodies (51).

aHUS is a complement-mediated TMA caused by the overactivation of the AP as a result of inherited and/or acquired complement abnormalities (52, 53). aHUS mainly targets the kidneys, and 50-70% of patients develop end-stage kidney disease (ESKD) unless they receive early and appropriate treatment (52). The efficacy and safety of the complement-inhibiting drug, eculizumab (54, 55) and ravulizumab (56, 57), have been described in many reports. Genetic variants of several complement regulators (FH, FI, and MCP) and activators (C3 and FB) have been identified in up to 50% of patients with aHUS (58). Thus, complementary diagnostic approaches have been developed to address the limitations of comprehensive gene analysis (59, 60). In addition, the acquisition of inhibitory autoantibodies against FH can cause aHUS (52, 61). In both genetic and acquired defects, impaired complement control on self-cell surfaces *via* the formation of C5b-9 leads to endothelial tissue damage and the generation of thrombi in the microvasculature (62).

Thrombocytopenia is a typical feature of TMA in which platelet thrombi are found in the capillaries and arterioles (62, 63). As mentioned above, various complement regulators normally protect platelets from complement attack; thus, defects in these regulators in aHUS may cause platelet activation and the formation of platelet-rich microvascular thrombi. Although there are few reports regarding the coagulation profile of aHUS (64), genetic variants of some coagulation-related proteins are associated with the pathogenesis of aHUS (65–67). Because the activations of complement and coagulation systems synergistically amplify each other as discussed earlier, this vicious cycle may be associated with the formation of microvascular thrombi in aHUS.

Patients with *FH* variants have a significantly increased risk of ESKD than those with variants in *CD46* (*MCP*) (68). *FH* variants associated with aHUS are primarily located at the C-terminus, and inhibitory autoantibodies also target the C-terminal region of FH (68–70). These genetic and acquired defects inhibit FH binding to the cell surface, resulting in C5b-9 formation in endothelial cells and platelets (62, 71). Although it is still unclear why complement damage seems to be restricted to the kidneys, heparan sulfate expressed in the kidneys appears critical for FH binding on cell surfaces (72).

In addition to TTP, STEC-induced HUS, and aHUS, a variety of pathological conditions such as autoimmune disease (73, 74), drug use (75), infection (76, 77), malignancy (78), malignant hypertension (79–81), pregnancy (82, 83), and transplantation (84, 85) can trigger secondary TMA. Although TTP and STEC-HUS have well-established diagnostic tests, the differentiation between aHUS and secondary TMA remains controversial because pathogenic variants in complement-related genes are only identified in about half of the patients with aHUS. In addition, complement abnormalities have been found in some secondary TMA (86).

Complement abnormalities may play a role in secondary TMA caused by malignant hypertension (79–81) and pregnancy (82). Rare genetic variants in complement-related genes have been identified in approximately 30-70% of patients with malignant hypertension-associated TMA (79–81). In these cases, *ex vivo* analysis showed that patient sera induced massive C5b-9 formation in microvasculature endothelial cells, suggesting that these variants induced excessive complement activation. In addition, patients with over-deposition of C5b-9 in vascular endothelial cells had a higher incidence of ESKD, and the administration of anti-C5 antibodies improved renal function in these cases.

### Lupus nephritis

Systemic lupus erythematosus (SLE) is a autoimmune disease that primarily affects young women. SLE affects the kidneys in approximately 50% of such patients as lupus nephritis (LN) (87). A variety of abnormal immune responses, such as defects in the clearance of immune complexes and apoptotic cells, nucleic acid-sensing abilities, lymphocyte signaling, and interferon-production have a central role in the pathogenesis of this disease (88, 89). Because immune complexes are deposited in tissues, and the deposition of C1q in tissues is relatively characteristic of SLE, it is plausible that these immune complexes activate the complement system and decrease complement levels (C3, C4, and CH50) in the peripheral blood. Activation of the complement system may cause inflammatory injury to tissues, as C3 or C4 fragments bind to complement receptors on B lymphocytes to enhance antibody generation (90, 91). However, this cannot explain why the deficiency of complement components such as C1q, C1s, C1r, C2, and C4 causes lupus-like symptoms (92). Deficiencies in this component activity may disturb the efficient disposal of dying and dead cells (93) or the normal tolerance mechanisms of lymphocytes (94), with both leading to the generation of autoantibodies.

The International Society of Nephrology/Renal Pathology Society meeting report mentioned the importance to have a standardized approach and terminology to distinguish ordinary arterial or arteriolar sclerosis from lupus-related lesions such as vasculopathy associated with immune complex deposition, vasculitis, and TMA (95). Indeed, within the glomerular capillaries and small arterioles in LN kidney specimens, microvascular thrombosis is conventionally recognized as one of the most common histopathological findings (96, 97). This characteristic finding has been variously described as “lupus vasculopathy (LV)” (97, 98), “immunoglobulin microvascular cast” (96), and “glomerular thrombosis” (99). LV is documented predominantly in diffuse proliferative LN, but the underlying etiology and its prognostic value remain undetermined (96, 97, 100). LV is characterized by the accumulation of immunoglobulins and complements in the vascular wall (98). This results in luminal narrowing and suggests the presence of immune-mediated vascular injury (98). Notably, unlike lupus vasculitis, LV does not involve the deposition of inflammatory cells in the vascular wall (98).

In lupus-prone MRL/lpr mice, treatment with aspirin and dexamethasone partially attenuates glomerular thrombosis (101). In rodents with nephrotoxic nephritis that mimics the downstream effector phase of LN (102), Fcγ receptors (103, 104) and components of the complement system (105, 106) have been implicated in the pathogenesis of tissue injury, including glomerular thrombosis. These experimental findings in animals suggest that inflammation may promote kidney glomerular thrombosis in LN.

Antiphospholipid syndrome (APS) frequently occurs in SLE and is characterized by vascular thrombosis, repeated miscarriages, and the presence of antiphospholipid antibodies (APLA) (107). Although patients with APS show a prolonged activated partial thromboplastin time, APLA exert prothrombotic effects because they inhibit β2-glycosylphosphatidylinisotol, which is an inhibitory regulator of phospholipid-dependent coagulation, protein C activation, thrombomodulin, and heparan sulfate on vascular endothelial cells (108, 109). Intriguingly, C4 deficiency attenuates fetal loss in mice with APS (110). Moreover, APLA stimulate TF activation in myelomonocytic cells, and mice deficient in C3, unlike mice deficient in C5, are protected from *in vivo* thrombus formation induced by cofactor-independent APLA, suggesting that C3 is required for TF activation and APLA-induced thrombosis (111). Because complement mediates TF enrichment in NETs (47, 48), neutrophils may be another player in thrombin formation in this context.

### Primary glomerulonephritis

Glomerulonephritis refers to a group of kidney diseases affecting the glomeruli due to the damage mediated by immunological dysregulation. Hypocomplementemia is a significant feature of kidney glomerular diseases such as post-infectious glomerulonephritis, immune complex-mediated membranoproliferative glomerulonephritis, C3 glomerulopathy, dense deposit diseases, and IgG4-related kidney disease (112, 113). In addition, the majority of patients with IgA nephropathy (114) or membranous nephropathy (115) have local C3 deposits in the glomeruli. Other complement elements, such as the FH-related proteins 1 and 5 and lectin pathway products, also accumulate (114–116). These findings suggest that systemic or local activation of the complement system may cause kidney tissue damage.

However, thrombosis is rarely observed in these patients unless they develop heavy proteinuria (117), which evokes a hypercoagulable state due to the urinary loss of coagulation regulatory proteins, including antithrombin and protein S, which counterbalances the increase in the synthesis rate of hemostatic proteins in the liver (118). Further studies are needed to determine the association of the development of thrombosis with complement-mediated kidney injury.

## Conclusion

The complement system has long been recognized as a central mediator of innate immune defenses that eliminate invading pathogens. Accumulating evidence has revealed how the complement system can modulate the function of the coagulation system, platelets, and neutrophils, and contribute to thrombosis. Further research on the link between the complement system and kidney disorders may deepen our understanding of complement-dependent mechanisms that promote glomerular capillary thrombosis and severely impair glomerular filtration. Such insights may provide novel therapeutic options for patients and clinicians.

## Author contributions

YY drafted the original manuscript and HN revised it. Both authors approved the submitted version of the manuscript.

## Funding

This work was supported by the Japan Society for the Promotion of Science Grant-in-Aid for Scientific Research 21K08272 (to YY) and the SENSHIN Medical Research Foundation (to HN).

## Acknowledgments

The figures were created with BioRender.com.

## Conflict of interest

The authors declare that the research was conducted in the absence of any commercial or financial relationships that could be construed as a potential conflict of interest.

## Publisher’s note

All claims expressed in this article are solely those of the authors and do not necessarily represent those of their affiliated organizations, or those of the publisher, the editors and the reviewers. Any product that may be evaluated in this article, or claim that may be made by its manufacturer, is not guaranteed or endorsed by the publisher.
